# Production of gas diffusion layers with cotton fibers for their use in fuel cells

**DOI:** 10.1038/s41598-022-08124-0

**Published:** 2022-03-10

**Authors:** A. J. Navarro, M. A. Gómez, L. Daza, J. J. López-Cascales

**Affiliations:** 1 Dep. Ing. Quimica y Ambiental, Universidad Politécnica de Cartagena, Campus Alfonso XIII, Aulario C, 30203 Cartagena, Murcia Spain; 2 Instituto de Catálisis y Petroquímica, C/ Marie Curie 2, Campus Cantoblanco, 28049 Madrid, Spain

**Keywords:** Energy science and technology, Materials science

## Abstract

The gas diffusion layer (GDL) is one of the most important parts of a proton exchange membrane fuel cell, that plays a key role transporting the current to the collector plates, distributing the reactant gases to the catalyst surface, and evacuating heat and water that is generated during the redox reactions inside the fuel cell. Speaking in terms of production cost, the GDL represents up to 45% of the total cost of the membrane electrode assembling (MEA). However, and despite its crucial role in a fuel cell, until recent years, the GDLs have not been studied with the same intensity as other MEA components, such as the catalyst or the proton exchange membrane. In this work, we present the production process, at laboratory scale, of a low cost GDL, using a non-woven paper-making process. A relevant aspect of this GDL is that up to 40% of their composition is natural cotton, despite which they present good electrical and thermal conductivity, high porosity, good pore morphology, high hydrophobicity as well as gas permeability. Furthermore, when the GDL with its optimum cotton content was tested in a single open cathode fuel cell, a good performance was obtained, which makes this GDL a promising candidate for its use in fuel cells.

## Introduction

During the last decades, hydrogen fuel cells have received growing interest due to an increasing concern about environmental pollution, and the successive international crises related to fluctuations in the market of the fossil fuels^1,2^. Thus, major projects are underway for developing a wide variety of stationary and mobile applications, that goes from stationary power backups to fuel cell vehicles, among others. In this context, fuel cells are electrochemical devices in which the electrical power is produced by hydrogen oxidation and oxygen reduction, generating only water and heat as products in the process^3–5^. The proton exchange membrane fuel cell (PEMFC) is the most widely used because they employ a solid and stable electrolyte, provide high current densities, request a short starting time, and keep a reasonable long lifetime^4,6,7^. An open cathode proton exchange membrane fuel cell (OC-PEMFC) is a type of these fuel cells in which oxygen is taken from the air using an external fan for producing air convection on the cathode^8–10^. The main advantage of this type of fuel cell compare with others is that they avoid the use of expensive peripheral devices that introduces severe penalties during their performance^10^.

In this context, the gas diffusion layer (GDL) constitutes one of the main components at the heart of the fuel cells, together with the catalyst and proton exchange membrane^4^. Thus, GDLs play a crucial role in collecting the electrons generated during the electrochemical reaction, they distribute the reactants to the catalyst surface, and they evacuate water and heat generated inside the fuel cell during the electrochemical reactions^11,12^.

However despite those important roles played by the GDLs, for years relatively low attention has been paid on this fuel cell component, compared with the effort put into the development of new catalysts and proton exchange membranes^4^. Fortunately, that trend has begun to change during the last years. Thus, for example, Lin et al.^13^ focused their effort on the effect of the presence of carbon nanotubes in the microporous layer (MPL) on the performance of a proton exchange membrane fuel cell. Chen et al.^14^ optimized the interface between the microporous layer and the electrolyte membrane with decorative patterns. Indayaningsih et al.^15^ produced new gas diffusion layers using carbon coconut fibers as the main ingredient for their preparation. Chen et al.^16^ showed as the use of ammonium chloride improved the porous structure of the MPL. Thus by recrystallization and pyrolysis of ammonium chloride with different content, the surface of an MPL exhibited a point, line and flower-like pattern, that improved its electrochemical response. Ji et al.^17^ showed the delicate equilibrium between the membrane drying and water flooding in a fuel cell. Ferreira-Aparicion et al.^18^ studied the effect of the gas diffusion layer at the cathode structure on the performance of an air-breathing proton exchange membrane fuel cell, where those authors found that the ability of the GDL macroporous structure to expel water from the cathode is a critical aspect for obtaining high-performance air-breathing fuel cells. Kim et al.^19^ studied the effect of the cracks in a gas diffusion layer on the performance of a fuel cell, and they obtained that the thinner the flow channels are, the GDL cracks affect more severely to the PEMFC performance. Zenyuk et al.^20^ studied the porosity, tortuosity, and pore-size distribution (PSD) under compression using X-ray for a set of commercial GDLs. Oualid et al.^21^ studied the effect of clamp pressure on the structure of a gas diffusion layer and its effect on the fuel cell performance. This paper describes an experimental method for measuring the electrical contact resistance versus the static mechanical pressure applied to the GDLs, finding a nonlinear behavior of the electrical contact resistance versus the mechanical stress is observed, where the addition of PTFE and MPL modify very severely the electrical contact resistance. Yang et al.^22^ studied the use of graphene oxide for fabricating gas diffusion layers. Those authors found that compared with standard carbon papers, the presence of graphene oxide (GO) in the carbon paper increased of carbon yield and crystallinity of matrix carbon, improving its tensile strength and electrical conductivity since more covalent bonds (C=O and C–O) formed between the GO and carbon fiber. Lee et al.^23^ proposed the employment of an improved single layer as gas diffusion layer in a fuel cell. They saw an increase in the roughness of its surface after graphene incorporation to its composition, which then, made gas distribution much easier. Tabe et al.^24^ studied the impact of the microporous layer morphology on the liquid water distribution at the catalyst interface and how this affects the fuel cell performance, finding that the MPL suppresses water accumulation at the interface due to smaller pore size and finer contact with the catalyst layer, avoiding the water flooding. In line with this study, Chen et al.^25^ investigated the effect of PTFE on the water transport in a gas diffusion layer, where those authors found that fuel cells with a 10wt % of PTFE in their GDL improved performance. Fadzillah et al.^26^ modeled the microstructure of a gas diffusion layer and analyzed the effect of hydrophobicity, thickness, porosity, and fiber diameter on the fuel cell performance. In this context, a type of GDL that is widely used in fuel cells is based on non-woven carbon paper, in which different components, such as carbon nanotubes and cellulose fibers coated with conducting polymers, have been added to the carbon paper during its fabrication to improve its electrical conductivity and permeability^27–31^.

In this work, we present a novel method of preparing a gas diffusion layer in which renewable cotton cellulose was used for its fabrication. The reason for choosing this natural fiber is because this is a cheap fiber, which is easy be found around the world. Finally, these GDLs were subsequently characterized and their performance studied in a single fuel cell.

## Experimental

### GDL preparation

The GDLs are usually composed of two different layers: the MPS (Macro Porous Substrate) and the MPL. Thus, the MPS provides mechanical support to the GDL and excellent electrical conductivity and gas permeability, while the MPL with its microporosity facilitates the gas distribution to the active sites on the catalyst layer^4,12,32^.

**Figure 1:**
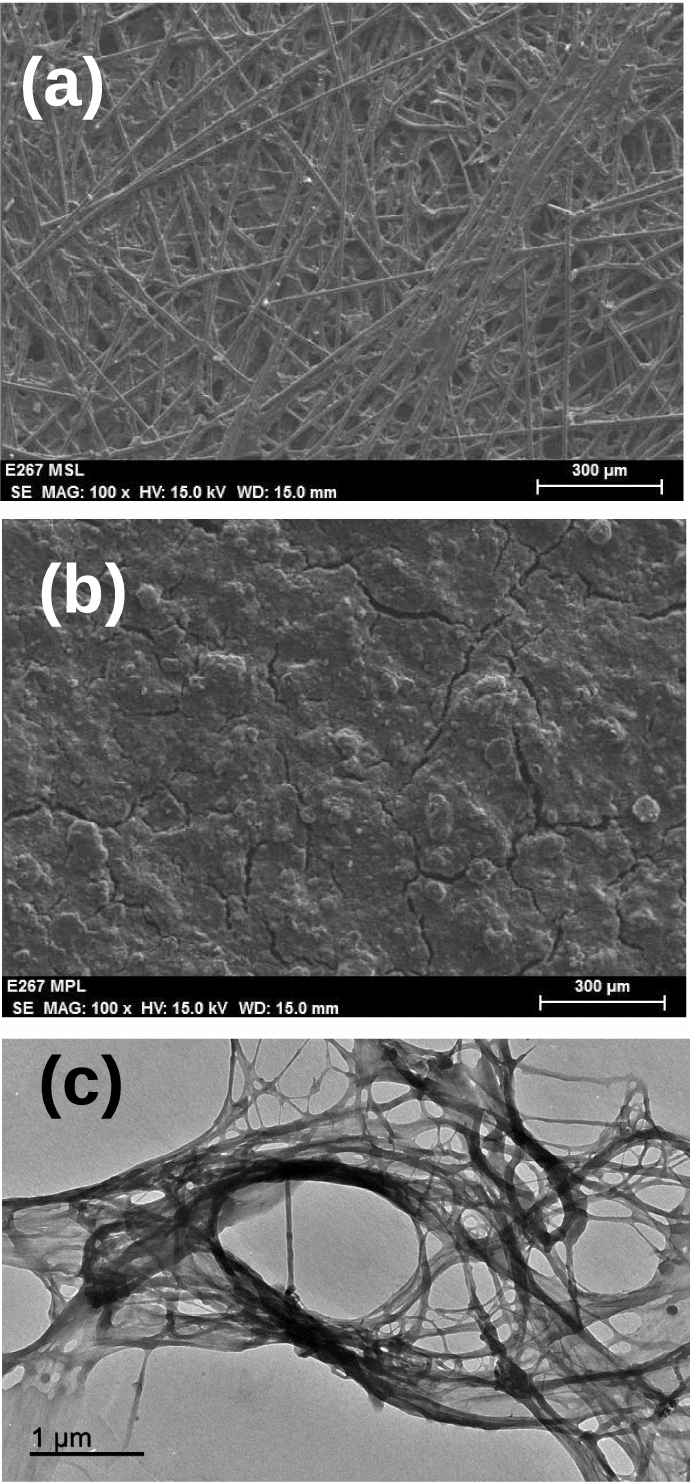
SEM images of the MPS (**a**) and MPL (**b**) of the GDL with a 40% in cotton fiber. TEM image of the natural cotton fibers used in this work (**c**).

The MPS was prepared with the following materials: carbon fibers of 3mm length (CF) (SGL-carbon), natural cotton fibers (CC) (see Figure 1), graphite powder (GP)( Merk), commercial polyamide epoxy (PE), Poly(tetrafluoroethylene) (PTFE) in aqueous solution 60 wt% (Alfa Aeser) and acetylene carbon black (AC) (Alfa Aesar).

In this process, several problems were faced at the beginning of this study, such as an inhomogeneous distribution of the graphite powder in the MPS, poor mechanical properties of the MPS after the thermal treatment, and so on. Thus, after several trials and attendant error in which a systematic variation of all the components was carried out, the final recipe for fabricating the MPS was as follows: 1.8 g CF, 1.1 g GP, and 6 ml PE. Thus, maintaining the quantities of these components constant, the cotton fiber was varied from 0.5 to 2 g, which represents a change from 14 to 40% of the total mass. Then, all those components were mixed in 1L of water and stirred for 20 min until a homogeneous suspension was obtained. Later, that supension was then filtered in a 170 mm diameter Büchner funnel, and the paste obtained after filtration was compacted with a pressure of 18 $\hbox {kg/cm}^2$. Finally, the paste was baked for 14 h at 200 $^{\circ }\hbox {C}$, followed by 25 min at 1000 $^{\circ }\hbox {C}$. At the end of this process, an MPS of $0.30\pm 0.05$ mm thick was obtained.

With respect to the MPL, it was generated by manual spraying deposition of an ink containing 0.5 g carbon black in 100ml of 2-isopropanol. This carbon solution was deposited on the MPS until a surface concentration of $1.7\,\hbox {mg/cm}^2$ carbon black was obtained. This process generated an MPL of around 0.12 mm thick. Thus, considering the MPS+MPL, a GDL of $0.42\pm 0.05$ mm was generated.

Figure 1 shows the morphology of the MPS and MPL generated following the procedure described above. Note that the small slits on the MPL surface are similar to those seen on the MPL of other commercial GDLs^28,33^, which are due to the stress generated during the solvent evaporation.

To increase the hydrophobicity of the GDL, it was sprayed with 10 ml of a solution of 2-isopropanol containing PTFE, until the MPL was coated with 25% PTFE, for an optimal response of the GDL in a fuel cell^34^.

### Electrode preparation

The electroactive electrodes were prepared by painting a thin film of catalyst on the proton exchange membrane of Nafion NR-212 (Ion-Power) using the electrospray technique^35–38^. Thus, an ink containing 0.04 g of 20% Pt on graphitized carbon (Sigma-Aldrich), and 0.002g of 5% Nafion solution (Alfa-Aesar), which represents 25% of the catalyst in weight was prepared. Both, Pt/C and Nafion were mixed in 40 ml of 2-isopropanol, subjected to ultrasound for 1 hour, and stirred for 24 h before use. After the ink was ready, the catalyst was deposited on the membrane till obtaining a concentration of $0.2\,\hbox {mgPt/cm}^2$.

### Single fuel cell

The fuel cell used for testing the GDLs was a single open cathode fuel cell (OC-PEMFC), and its plates were manufactured in our university using 304 stainless steel. Later, both plates were covered with a very thin layer of gold to improve their electrical conductivity and electrochemical stability. The anode was designed considering parallel channels of 2 mm width and 1 mm depth, with 1 mm rib width, while the cathode was designed by parallel channels of 2 mm width, 3 mm depth, and 1 mm rib width. In this fuel cell, the cathode was open to the air at atmospheric pressure, and oxygen was blown to the fuel cell using an external fan, coupled to the fuel cell. To prevent the proton exchange membrane from drying, the cathode channels were maintained parallel to the table surface, and the fan speed was controlled electronically as a function of the required current. The temperature was initially fixed at 40 $^{\circ }\hbox {C}$, and hydrogen was used directly from the bottle without humidification, with a dead back pressure of 1 bar. The air temperature and humidity were maintained at 30 $^{\circ }\hbox {C}$ and 50–55% RH, respectively, through a standard air conditioning unit. The active surface area of this single fuel cell was $23.1\,\hbox {cm}^2$.

## Results and discussion

### Polarization curves

**Figure 2:**
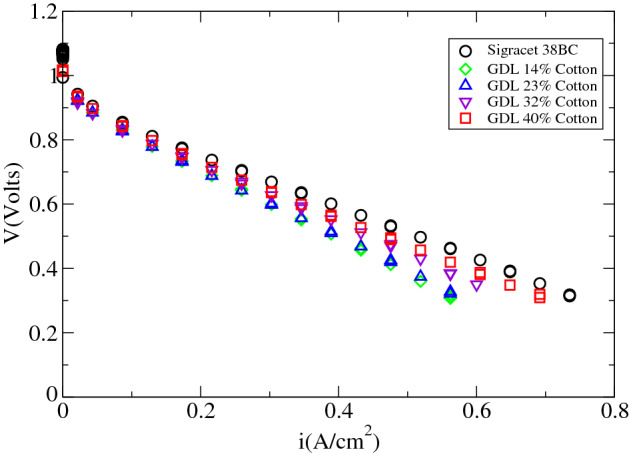
Polarization curves with gas diffusion layer of different cotton fiber content and Sigracet 38BC.

Figure 2 shows the polarization curves of all the GDLs generated in this study, together with the commercial Sigracet 38BC (SGL-carbon). The polarization curves were generated using an electronic DC Load 3721A of Array Electronic Co., Ltd. (http://www.array.sh/). Those polarization curves were attained at 40 $^{\circ }\hbox {C}$ after 7 days since activation, working at a constant current density of $0.2\,\hbox {A/cm}^2$. To prevent an excess of water accumulation in the anode, hydrogen was purged every 30 min, with a purge time of 0.2 s. Figure 2 shows how our GDL with 40% of cotton provides similar performance to Sigracet 38BC. This difference increases with the cotton fiber content in our GDls. In a first instance, the variation in their through-plane electrical conductivity, and porosity, can justify the worsening in their performance with the diminishing in the cotton fiber content, as discussed below.

The relation between cell potential and current density has been shown to obey to the following equation^39^,

1 $$\begin{aligned} E=E_0-b\log i-R.i \end{aligned}$$

where,

2 $$\begin{aligned} E_0=E_r-b\log i_0 \end{aligned}$$

being $E_r$ the reversible potential, $i_0$ the exchange current, *b* the Tafel slope, and R the lineal resistance that gathers the contribution of the proton exchange membrane and Ohmic resistances. Table 1 shows the fitting parameters of the polarization curves of Fig. 2. Thus, Table 1 shows how the fuel cell with Sigracet 38BC presents an Ohmic resistance similar to the ours, when the GDLs fiber content is above 32%. Table 1 shows the diminishing of the Ohmic resistance with the cotton fiber content.

**Table 1:** Fitting parameters of the polarization curves of Fig. 2 using Eq. (1).

GDL	$E_0$ (V)	$b\,(\hbox {V}\,\hbox {dec}^{-1}$)	$R\,(\Omega \,\hbox {cm}^2$)
38BC	0.84	0.049	0.88
14% cotton	0.91	0.020	1.06
23% cotton	0.87	0.039	0.098
32% cotton	0.87	0.039	0.086
40% cotton	0.86	0.037	0.083

### Electrical conductivity

A gas diffusion layer presents an anisotropic structure due to the different orientation of its components in- and through- the plane. Hence, two electrical conductivities have to be specified to characterize its electrical conductivity. The electrical conductivity in the plane was measured using the linear four points method, with a distance of 5mm between two consecutive points.This method consists in determining the potential difference between two points due to the application of an electrical current between the other two, maintaining constant the distance between consecutive points^40,41^. Thus, the electrical resistivity in the plane can be measured as follows^40^:

3 $$\begin{aligned} \rho _{in}=\frac{\pi t}{\ln 2}\frac{V}{I} \end{aligned}$$

where t is the sample thickness, I the electrical current applied between the two external points and V the electrical potential measured between the other two points. Hence, the electrical conductivity, $\sigma _{in}$, can be obtained as follows,

4 $$\begin{aligned} \sigma _{in}=\frac{1}{\rho } \end{aligned}$$

where $\sigma _{in}$ is expressed in S/m.

**Figure 3:**
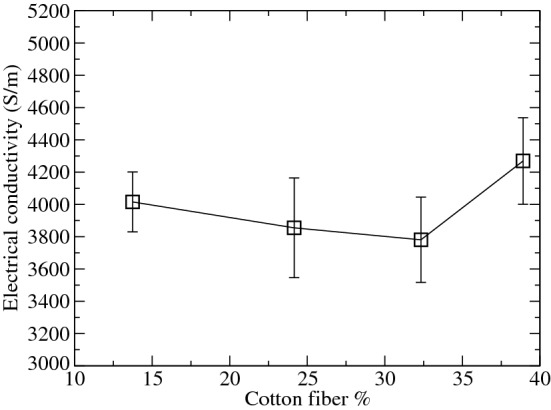
In-plane electrical conductivity of the GDLs with different percentages of cotton fiber. Error bars were determined from three different samples.

In this regard, Fig. 3 shows the variation of the electrical conductivity corresponding to GDLs with different cotton content.

Figure 3 shows how the electrical conductivity remains almost constant for all the GDLs, after discarding the error bars. Thus, the electrical conductivity corresponding to the GDL with a 40% cotton is similar to the value of $\sigma _{in} = 4421\pm 160$ S/m measured by Ozden et al.^4^ for the commercial Sigracet 38BC.

**Figure 4:**
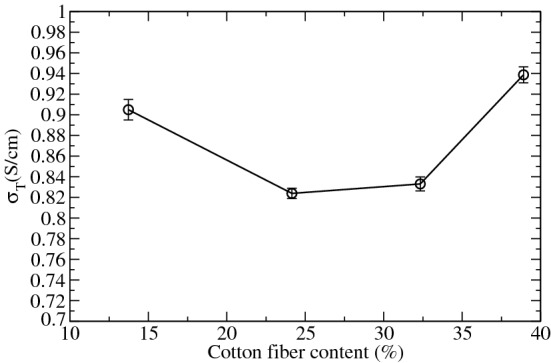
Through plane electrical conductivity of the GDLs with different percentages of cotton content. Error bars were determined from three different samples.

The through-plane electrical resistivity, $\rho _T$, was obtained from equation 5 (^42^):

5 $$\begin{aligned} R_T=\frac{\rho _T.l}{A} \end{aligned}$$

where $R_T$ is the electrical resistance of a sample confined between two copper plates with a golden bath, under a pressure of $10\,\hbox {kg/cm}^2$ and measured with a miniOhmeter BK-Precision BA6010 at 1 kHz, $\rho _T$ is the resistivity expressed as $\frac{\hbox {Ohms}\,\hbox {cm}^2}{\hbox {cm}}$, *A* is the transversal surface, and *l* the sample thickness. Thus, the through plane conductivity, $\sigma _{T}$, corresponds to the inverse of $\rho _T$, $\sigma _T=\frac{1}{\rho _T}$, and its units are S/cm. Figure 4 shows the through-plane electrical conductivity of the GDLs with different cotton content.

Figure 4 shows how this electrical conductivity diminish when cotton fiber content increases from 14 to 23%, remaining constant till 32%, and increasing for 40% cotton content. Thus, an increase in the cotton content produces a diminishing in the through-plane electrical conductivity till reaching a critical value, where beyond that point, the electrical conductivity rises. The explication of this behavior is in the sense that beyond a certain critical cotton content, an increase in the number of electrical contacts through the plane is expected as a consequence of the fact that more graphite powder and carbon fibers fall trapped between the cotton fibers during the filtration process, and hence, an increase of the electrical conductivity through the plane is expected. Those values measured for our GDLs are typically a 50 % of the value for the Sigracet 38BC, where $2.00\pm 0.04$ S/cm was measured.

With the aim of determining the variation of the through-plane conductivity with pressure, Table 2 shows the through-plane conductivity of a GDL with 40% cotton content in a wide range of pressures, in comparison with the Sigracet 38BC.

**Table 2:** Through-plane electrical conductivity as a function of pressure for the GDL with 40% cotton fiber and Sigracet 38BC.

Pressure $(\hbox {kg/cm}^2)$	31	45	76	106
40% cotton (S/m)	0.938	1.63	2.63	3.33
Sigracet 38BC (S/m)	2.0	3.9	3.7	3.6

From the results of Table 2, the electrical conductivity of the GDL with a 40% cotton content converges to the values measured for the Sigracet 38BC only at very high pressures. However, it is of especially relevance that the thickness of the GDL with 40% cotton content was reduced scarcely a 30% under a pressure of $106\,\hbox {kg/cm}^2$, against the 50% of the Sigracet 38BC, under the same pressure.This increase in the compression of the Sigracet may play an important role in reducing the pore morphology, i.e the porosity of the GDLs at high clamping pressures.

### Porosity and hydrophobicity

**Figure 5:**
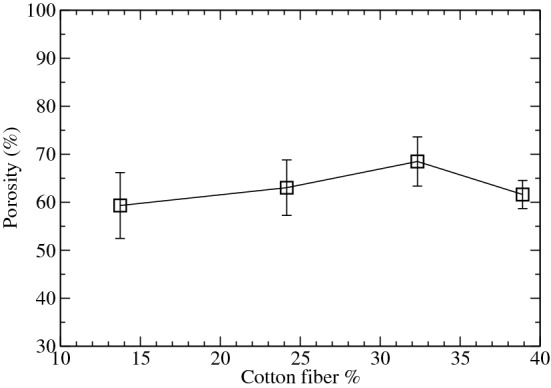
Porosity of the GDLs depending on the cotton fiber content. Error bars were determined from three different samples.

Porosity and hydrophobicity provide information on the ease with which gases can penetrate the GDL and how the GDL deals with the water during the electrochemical reactions inside the fuel cell. To measure the porosity of the GDLs, in a first instance, the pycnometer method was employed for its determination, using kerosene (Sigma-Aldrich) as solvent. The porosity $\varepsilon$ s defined as the ratio between the void volume of the sample ($V_v$) and the total volume of the sample ($V_s$), including air. Thus, the porosity $\varepsilon$ was measured as follows^43^:

6 $$\begin{aligned} \epsilon = \frac{V_v}{V_s} \end{aligned}$$

where $V_v$ is measured using kerosene as solvent at 25 $^{\circ }\hbox {C}$, and $V_s$ is the macroscopic volume of the sample. Figure 5 shows how the porosity increases with the cotton fiber content. But this trend is broken when cotton reached a 40% content. But this trend is broken when cotton reached a 40% content. This behavior is explained by the fact that cotton fibers have a branched structure (see Fig. 1) which increases the empty space in the GDL due to fiber overlaps. This result is in perfect agreement with the electrical conductivity measured above, in which the GDL with a 40% cotton content showed the highest conductivity since more graphite powder is trapped between the fibers that form the MPS during its production, and as a consequence, the electrical conductivity increases, i.e. its porosity diminishes, in a perfect correlation between both properties. In general, all our GDLs showed a porosity above 60%.

To provide further insight into the morphology of those GDLs, the pore distribution and porosity of the GDLs were studied using the mercury porosimetry technique. Thus, the porous distribution was determined using the intrusion-extrusion mercury porosimeter Autopore IV 9510 located at the Institute of catalyst and Petrochemical, CSIC, Madrid. The pore size distribution was determined considering the volume of mercury that penetrates the samples as a function of the pressure. Washburn equation Equation, Ecuation 7,was applied for obtaining the pore size distribution considering a pore cylindrical shape. Thus, the pore size distribution was calculated as follows:

7 $$\begin{aligned} d=-\frac{4\sigma \cos \theta }{p} \end{aligned}$$

where $\theta =130^0$, and $\sigma =485\,\hbox {dyn}\,\hbox {cm}^{-1}$ for mercury.

**Figure 6:**
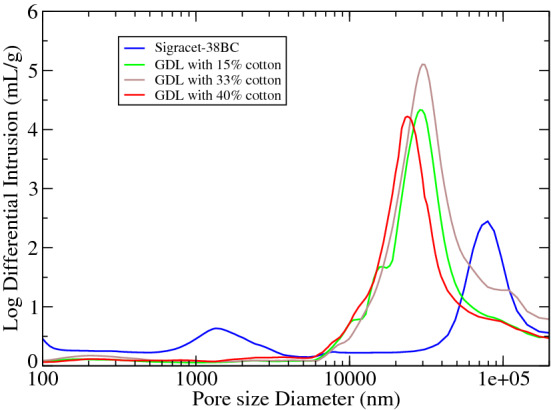
Pore distribution determined by mercury porosimetry technique.

Figure 6 shows a mono-modal distribution for our GDLs, with a maximum at 28,000 nm that contrasts with the bimodal distribution of the Sigracet 38BC with two peaks at 1384 and 78,000 nm respectively, with an average pore diameter of 442.4 nm for the Sigracet 38BC in comparison with the diameter of 254 nm for our GDL with 40% cotton content. This diminishing in the pore diameter for the GDL with 40% cotton fiber is crucial for a proper water management in the interior of the fuel cells, since pore diameter is involved in the water condensation process, according to the Young-Laplace equation, for very hydrophobic surfaces. Thus, Table 3 shows a summary of the most relevant pore parameters of our GDLs and Sigracet 38BC.

**Table 3:** Porosity, pore area and pore diameter determined by mercury porosimetry.

Property	GDL 14% cotton	GDL 32% cotton	GDL 40% cotton	Sigracet 38BC
Porosity (%)	66.2	59.29	69.98	74.27
Pore area (59917 psia) m$^2$/g	18.190	8.010	37.006	16.664
Pore diameter (4V/A) (nm)	516.93	148.46	254.85	442.4

From the results of Table 3, we observe as in general, Sigracet 38BC presents a porosity that is 5.7% higher than the GDL with a 40% cotton content. Thus, Sigracet 38BC shows a porosity of 74.27% that is slightly below to the value of 80% indicated in its technical data sheet. In our case, the porosity measured using the mercury porosimetry technique is roughly 15% higher than the values obtained using the pycnometer method described above. Furthermore, Fig. 6 shows a shift in the pore distribution toward lower values in their pore diameter with the increase in cotton fiber, which can be justified on the basis that more graphite powder is trapped between the fibers during the MPS filtration process, and as a consequence, a diminishing in the maximum of the pore distribution toward lower values is expected.

Thus, hydrophobicity together with the porosity of the GDLs (properties that are related to each other^44^) are two key properties for managing the water generated in a fuel cell^33^. An estimation of the contact angle of a water droplet of 30 micro-liters with the MPL was carried out, obtaining angles of 170° and 152° for our GDL with 40% cotton content and Sigracet 38BC, respectively.

### Air permeability

Although air permeability is in part related to its porosity, this assessment is only partially true because permeability is a property that is narrowly associated with the morphology of the pore distribution, while porosity is merely related to the void volume available inside the GDL. In our case, the permeability was measured using the Gürley method, such as it is described in the UNE Norm 57066-2:2003. The surface area through which air is forced to pass was $6.45\,\hbox {cm}^2$.

**Figure 7:**
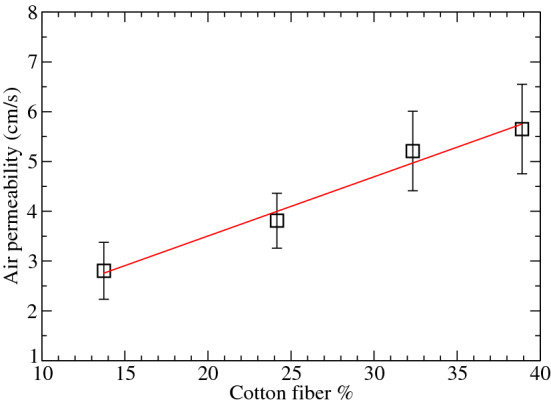
GDL permeability as a function of the cotton fiber content using the Gürley method. Error bars were determined from three different samples.

Figure 7 shows how the permeability to air increases with the cotton content. Such behavior is probably due to the fact that when more natural fibers are present in the GDL, the greater the number of connections between neighboring pores is expected and hence, the permeability to gases increases. Given that permeability measured for the Sigracet- 38BC was 1.2 cm/s, our GDLs showed an increase of roughly 80% with respect to Sigracet-38BC, even when Sigracet 38BC presents a porosimetry much higher than our GDL with 40% cotton content.

### Thermal conductivity, $\kappa$

The thermal conductivity, $\kappa$, was measured on the assumption that a flat thin surface separated two fluids (air) at different temperatures. Thus, under this premise, the thermal conductivity can be measured using a home-made chamber that is controlled electronically with Arduino (Arduino trademark, www.arduino.cc ), where $T^f_1$ corresponds to the temperature of the fluid (air) outside of the chamber, $T^S_1$ is the temperature at the outlet face of the GDL, $T^S_2$ is the temperature at the inlet face of the GDL, $T^f_2$ is the temperature of air inside the chamber, and b the GDL thickness.

Thus, in an stationary regime, we can write:

8 $$\begin{aligned} q=\frac{\phi }{S}=h_1(T^f_1-T^S_1)\longrightarrow T^f_1-T^S_1=\frac{q}{h_1}=\frac{1}{h_1}\frac{\phi }{S} \end{aligned}$$

In a stationary state, this power will be transmitted by conduction through the wall,

9 $$\begin{aligned} q=\frac{\lambda }{b}(T^S_1-T^S_2)\longrightarrow T^S_1-T^S_2=\frac{b}{\kappa }q \end{aligned}$$

and the same quantity from the surface $S_2$ to the cold fluid,

10 $$\begin{aligned} q=h_2(T^S_2-T^S_1)\longrightarrow T^S_2-T^f_2=\frac{q}{h_2} \end{aligned}$$

Thus, by combining the equations 9 and 10, the following expression is obtained:

11 $$\begin{aligned} \kappa =\frac{b.(T^S_2-T^f_2).h}{(T^S_1-T^S_2)} \end{aligned}$$

where the thermal conductivity $\kappa$ can be obtained from the measurement of four temperatures: the temperatures corresponding to the inside and outside of the isolated chamber, and the temperatures on both faces of the sample (GDL). Finally, h is a parameter that must be fitted using a substance of reference. In our case, we used as standard a piece of glass of 2 mm thick, where $1.4 \frac{\hbox {W}}{\hbox {K}\,\hbox {m}}$ was considered as the reference thermal conductivity^45^. To verify this procedure, the thermal conductivity of a piece of polypropylene film of 0.5 mm thick was measured, obtaining a value of $0.193\pm 0.004\, \frac{\hbox {W}}{\hbox {K}\,\hbox {m}}$ at 30 $^{\circ }\hbox {C}$, which is in perfect agreement with the reported data, which range from 0.17 to 0.22 $\frac{\hbox {W}}{\hbox {K}\,\hbox {m}}$^46,47^

**Figure 8:**
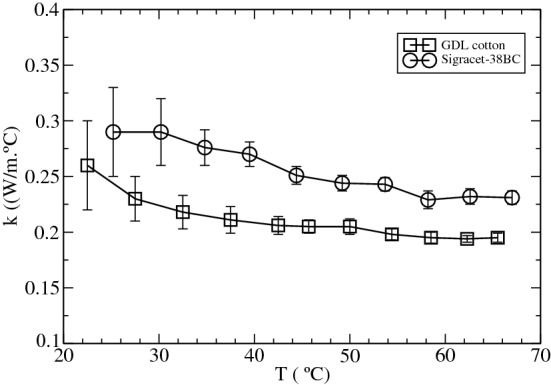
Thermal conductivity of a GDL with 40% cotton fiber content and Sigracet 38BC, as a function of temperature. Error bars were determined from three different samples.

After calibration, the thermal conductivity of the GDL with 40% cotton was measured as a function of the temperature, since this GDL was the GDL that showed the best electrical conductivity and permeability to gases, making of this the most suitable candidate for its use a fuel cell. Figure 8 shows the variation of $\kappa$ with temperature, for the GDL with a 40% fiber content and Sigracet 38BC.

Looking at Fig. 8, we see as Sigracet 38BC shows higher thermal conductivity than our GDL for the whole range of temperatures studied. For temperatures below 40 °C, Sigracet 38BC showed a thermal conductivity around 30% higher than the GDL with cotton. This difference is reduced to 20% for temperatures above 50 °C.

Thus, a value of $\kappa =0.195\pm 0.006\,\frac{\hbox {W}}{\hbox {K}\,\hbox {m}}$ and $0.229\pm 0.008\,\frac{\hbox {W}}{\hbox {K}\,\hbox {m}}$ was measured at 58 $^{\circ }\hbox {C}$ for the GDL with 40% cotton and Sigracet-38BC, respectively, which are in the same order of magnitude as the values of $\kappa$ reported for different Sigracet grades which ranged from $0.22\pm 0.04$ to $0.31\pm 0.06 \,\frac{\hbox {W}}{\hbox {K}\,\hbox {m}}$^48^.

## Conclusions

During recent decades, hydrogen has become a plausible alternative to fossil fuels, because when hydrogen is used in a fuel cell we are able to produce electrical current without emitting polluting gases, being only heat and water the products generated in the process.

The Gas Diffusion Layer (GDL) is one of the main components at the heart of the PEMFC. One of the main problems that constrain the spread of fuel cells in stationary and mobile applications is associated with the cost of the key components of these electrochemical devices: the catalyst, the proton exchange membrane, and the gas diffusion layer, where the GDL represents up to 45% of the total cost of an MEA fabrication, depending on the catalyst content.

In this work, we presented for the first time, a method for fabricating GDLs at laboratory scale with a high content of renewable material, in our case, natural cotton fiber. Thus, those GDLs were produced using an environmentally friendly method in which water was used as a solvent for preparing the macro porous substrate (MPS) instead of polluting and hazardous organic solvents.

Thus, after an ex-situ study of the electrical conductivity, porosity, permeability, and thermal conductivity, those GDLs showed a behavior that approaches to the shown by the commercial ones, although further studies have to be carried out for improving its performance in a fuel cell.

## References

[1] Busby RL (2005). Hydrogen and Fuel Cells.

[2] Srinivasan S (2007). Fuel Cells: From Fundamentals to Applications.

[3] Litster S, McLean G (2004). Pem fuel cell electrodes. J. Power Sources.

[4] Ozden A, Shahgaldi S, Li X, Hamdullahpur F (2019). A review of gas diffusion layers for proton exchange membrane fuel cell-with a focus on characteristics, characterization techniques, materials and designs. Prog. Energy Combust. Sci..

[5] Spiegel CS (2007). Designing and Building Fuel Cells.

[6] Borup R (2007). Scientific aspects of polymer electrolyte fuel cell durability and degradation. Chem. Rev..

[7] Cheng X (2007). A review of pem hydrogen fuel cell contamination: Impacts, mechanisms and mitigation. J. Power Sources.

[8] Huang Z, Su A, Hsu C, Liu Y (2014). A high-efficiency, compact design of open-cathode type pemfcs with a hydrogen generation system. Fuel.

[9] Wu B (2013). The performance improvement of membrane and electrode assembly in open-cathode proton exchange membrane fuel cell. Int. J. Hydrogen Energy.

[10] Atkinson R, Rodgers J, Hazard M, Stroman R, Gould B (2018). Influence of cathode gas diffusion media porosity on open-cathode fuel cells. J. Electrochem. Soc..

[11] Li H (2008). A review of water flooding issues in the proton exchange membrane fuel cell. J. Power Sources.

[12] El-Kharouf A, Rees N, Steinberger-Wilckens R (2014). Gas diffusion layer materials and their effect on polymer electrolyte fuel cell performance- ex situ and in situ characterization. Fuel Cells.

[13] Lin R (2020). Detailed optimization of multiwall carbon nanotubes doped microporous layer in polymer electrolyte membrane fuel cells for enhanced performance. Appl. Energy.

[14] Chen L (2020). Microporous layers with different decorative pattern for polymer electrolyte membrane fuel cells. Appl. Mater. Interfaces.

[15] Indayaningsih N, Zulfia A, Priadi D, Hendrana S (2016). Preparation if carbon composites from coconut fiber for gas diffusion layer. Ionics.

[16] Chen G, Zhang G, Guo L, Liu H (2016). Systematic study on the function and mechanism of micro porous layer on water transport in proton exchange membrane fuel cell. Int. J. Hydrogen Energy.

[17] Ji M, Wei Z (2009). A review of water management in polymer electrolyte membrane fuel cell. Energies.

[18] Ferreira-Aparicio P, Chaparro A (2014). Influence of the gas diffusion cathode structure on the performance of an air-breathing proton exchange membrane fuel cell. Int. J. Hydrogen Energy.

[19] Kim G, Kim D, Kim J, Kim H, Park T (2020). Impact of cracked gas diffusion layer on performance of polymer electrolyte membrane fuel cells. J. Ind. Eng. Chem..

[20] Zenyuk I, Parkinson DY, Connolly L, Weber A (2016). Gas-diffusion-layer properties under compression via x-ray tomography. J. Power Sources.

[21] Oualid S, Lachat R, Candusso D, Meyer Y (2017). Characterization process to measure the electrical contact of gas diffusion layers under mechanical static compressive loads. Int. J. Hydrogen Energy.

[22] Yang P, Xie Z, Li H, Wang P, Huang Q (2017). Graphene oxide reinforced ultra-thin carbon paper used for fuel cells and the mechanism of reinforcement. Int. J. Hydrogen Energy.

[23] Lee H, Chang J, Chen-Yang Y (2018). Improvement in physical properties of single-layer gas diffusion layers using graphene for proton exchange membrane fuel cells. RSC Adv..

[24] Tabe Y, Aoyama Y, Kadowaki K, Suzuki K, Chikahisa T (2015). Impact of micro-porous layer on liquid water distribution at the catalyst layer interface and cell performance in a polymer electrolyte membrane fuel cell. J. Power Sources.

[25] Chen Y (2018). Influence of ptfe on water transport in gas diffusion layer of polymer electrolyte membrane fuel cell. Int. J. Electrochem. Sci..

[26] Fadzillah D, Rosli M, Talib M, Kamarudin S, Daud W (2017). Review on microstructure modelling of a gas diffusion layer for proton exchange membrane fuel cells. Renew. Sustain. Energy Rev..

[27] Mathur R, Maheshwari P, Dhami T, Sharma R, Sharma C (2006). Processing of carbon composite papers as electrode for fuel cell. J. Power Sources.

[28] Isikel L, Gocek I, Adanur S (2010). Design and characterization of nonwoven fabrics for gas diffusion layer in polymer electrolyte membrane fuel cell. J. Text. Inst..

[29] Jabbour L, Chaussy D, Eyraud B, Beneventi D (2012). Highly conductive graphite/carbon fiber/celulose composite papers. Compos. Sci. Technol..

[30] Maheshwari P, Gupta C, Mathur R (2014). Role of fiber length and pore former on the porous network of carbon paper electrode and its performance in pemfc. Fuel Cells.

[31] Shi Y, Wang B (2014). Mechanical properties of carbon fiber/cellulose composite papers modified by hot-melting fibers. Prog. Nat. Sci. Mater. Int..

[32] El-Kharouf A, Mason T, Brett D, Pollet B (2012). Ex-situ characterisation of gas diffusion layers for proton exchange membrane fuel cells. J. Power Sources.

[33] Lapicque F, Belhadj M, Bonnet C, Pauchet J, Thomas Y (2016). A critical review on gas diffusion micro and macroporous layers degradations for improved membrane fuel cell durability. J. Power Sources.

[34] Xiong Z (2015). Enhanced water management in the cathode of an air-breathing pemfc using a dual catalyst layer and optimizing the gas diffusion and microporous layers. Int. J. Hydrogen Energy.

[35] Varea A, Monereo O, Xuriguera E, Prades J, Cirera A (2017). Electrospray as a suitable technique for manufacturing carbon-based devices. J. Phys. D Appl. Phys..

[36] Castillo J, Martin S, Rodriguez-Perez D, Higuera F, Garcia-Ybarra P, Garcia-Ybarra P (2018). Nanostructured porous coating via electrospray atomization and deposition of nanoparticle suspensions. J. Aerosol Sci..

[37] Bodnar E, Grifoll J, Rosell-Llompar J (2018). Polymer solution electrospraying: A tool for engineering particles and films with controlled morphology. J. Aerosol Sci..

[38] Yan J (2019). Highly conductives graphene paper with vertically aligned reduces graphene oxide sheets fabricated by improved electrospray deposition technique. Appl. Mater. Interfaces.

[39] Kim J, Lee S, Srinivasan S (1995). Modeling of proton exchange membrane fuel cell performance with an empirical equation. J. Electrochem. Soc..

[40] Miccoli I, Edler F, Pfnür H, Tegenkamp C (2015). The 100th anniversary of the four-point probe technique: The role of probe geometries in isotropic and anisotropic systems. J. Phys. Condens. Matter.

[41] Alarifi I (2019). Investigation the conductivity of carbon fiber composites focusing on measurement techniques under dynamic and static loads. J. Mater. Res. Technol..

[42] Cooper K (2011). Characterizing through-plane and in-plane ionic conductivity of polymer electrolyte membranes. ECS Trans..

[43] Ruggieri L, Gea T, Artola A, Sánchez A (2009). Air filled porosity measurements by air pycnometry in the composting process: A review and a correlation analysis. Bioresour. Technol..

[44] Cassie A, Baxter S (1944). Wettability of porous surfaces. Faraday Soc..

[45] Lide DR (2007). CRC Handbook of Chemistry and Physics.

[46] Birley A, Heath R, Scott M (1991). Plastic Materials. Properties and Applications.

[47] Maier C, Calafut T (1998). Polypropylene. The Definitive Users Guide and Databook.

[48] Kandelwal M, Mench M (2006). Direct measurement of through-plane thermal conductivity and contact resistance in fuel cell materials. J. Power Sources.

